# Connection between Weather Types and Air Pollution Levels: A 19-Year Study in Nine EMEP Stations in Spain

**DOI:** 10.3390/ijerph20042977

**Published:** 2023-02-08

**Authors:** Nuria Pardo, Samuel Sainz-Villegas, Ana I. Calvo, Carlos Blanco-Alegre, Roberto Fraile

**Affiliations:** 1 Department of Applied Physics, Faculty of Sciences, University of Valladolid, Paseo de Belén, 7, 47011 Valladolid, Spain; 2 Department of Physics, University of León, 24071 León, Spain; 3 IHCantabria-Instituto de Hidráulica Ambiental de la Universidad de Cantabria, 39011 Santander, Spain

**Keywords:** atmospheric pollution, EMEP, PM_10_, trends, weather types

## Abstract

This study focuses on the analysis of the distribution, both spatial and temporal, of the PM_10_ (particulate matter with a diameter of 10 µm or less) concentrations recorded in nine EMEP (European Monitoring and Evaluation Programme) background stations distributed throughout mainland Spain between 2001 and 2019. A study of hierarchical clusters was used to classify the stations into three main groups with similarities in yearly concentrations: GC (coastal location), GNC (north–central location), and GSE (southeastern location). The highest PM_10_ concentrations were registered in summer. Annual evolution showed statistically significant decreasing trends in PM_10_ concentration in all the stations covering a range from −0.21 to −0.50 µg m^−3^/year for Barcarrota and Víznar, respectively. Through the Lamb classification, the weather types were defined during the study period, and those associated with high levels of pollution were identified. Finally, the values exceeding the limits established by the legislation were analyzed for every station assessed in the study.

## 1. Introduction

The World Health Organization (WHO) states that suspended particulate matter (PM) is one of the atmospheric pollutants that produce the greatest effect on human health [1]. The concentration of aerosols depends on different variables, including weather conditions and atmospheric stability [2]. There are air quality studies based on meteorological conditions that show that the levels of the different pollutants vary depending on the weather types [3,4].

The transport of atmospheric pollutants acquires great relevance in air pollution studies, since the important role of meteorological phenomena comes into play. This transport is particularly evident in remote stations, located far from sources of anthropogenic pollution. The importance of these sampling points is highlighted through the creation of different programs focused on solving transboundary air pollution problems, among other tasks. Thus, in 1979, European authorities created the Convention on Long-Range Transboundary Air Pollution (LRTAP) [5]. This agreement addresses problems related to eutrophication and acidification, tropospheric ozone, heavy metals, persistent organic compounds, and PM. In Spain, the network with this mission is known as EMEP/VAG/CAMP related to AEMET (National Agency for Meteorology, in its Spanish acronym). The network is integrated by various programs: EMEP (European Monitoring and Evaluation Programme), VAG program (Vigilancia Atmosférica Global), and CAMP (Comprehensive Atmospheric Monitoring Programme).

Because fluctuations in atmospheric pollutant concentrations are closely related to meteorological parameters [6], the movement of air masses is a crucial aspect for the study of air pollution. Weather type classifications constitute an important approach for dealing with atmospheric circulation [7]. For this reason, including these classifications in the studies of atmospheric pollution transport is crucial. In recent years, several authors have published different automatic classification models for the Iberian Peninsula [8,9,10,11,12].

The circulation of the atmosphere can be studied from several approaches. One of them is the analysis of the weather types that affect a specific place at a specific time. The classification into weather types was initially based on the daily synoptic situation, using the shape of the isobars (which entails a certain level of subjectivity). However, currently, circulation is classified according to sea level pressure at several points close to the study area [10,13,14]. This type of classification has also been applied to the Iberian Peninsula [7,8,9,12,15,16,17], mostly in studies on precipitation.

In addition to purely meteorological studies, the classification into weather types has more recently been used to search for the contribution of atmospheric circulation to some pollution episodes [18], such as the Saharan dust intrusions in the Iberian Peninsula [4]. More recently, this classification has also been used, along with other techniques, for the control of aerosol pollution [19], for the search for sources and sinks of atmospheric aerosol [2,20,21] and for analyzing the influence of weather on health [22,23].

Long-term studies are of particular interest when continuous pollution data are available for long periods of time. With these studies, the effectiveness of national and international policies and regulations carried out to reduce levels of air pollution can be verified. So far, there are several studies that show a progressive decreasing trend in atmospheric pollutants, such as CO, NO_x_, SO_2_, O_3_, and PM. Most of those studies are focused on urban areas: Guerreiro et al. [24] focus on 38 European cities during the period 2002–2011, and Gualtieri et al. [25] study the trends of different pollutants in the city of Florence, Italy, between 1993 and 2012. Querol et al. [26] focus on different Spanish cities between the years 2001 and 2012. However, studies related to background regions are still scarce [27,28]. Two different PMs are usually studied, PM_10_ (PM with a diameter of 10 µm or less) and PM_2.5_ (PM with a diameter of 2.5 µm or less). Both of them are pollutants that can lead to major environmental and public health problems. The European directives and regulations indicate that a daily PM_10_ concentration of 50 µg m^−3^ (European Union Official Diary, Directive 2008/50/EC of the European Parliament and of the Council of 21 May 2008) should not be surpassed more than 35 days a year. The threshold applied in the PM_2.5_ case makes reference to a mean annual value of these concentrations instead of daily value. Although both pollutants have great relevance in air quality and atmospheric contamination studies, the analysis of PM_10_ concentrations is more suitable when the daily exceedances or the relationship between PM_10_ values and (daily) weather types needs to be studied since the threshold set by regulations includes also a daily value. In the context of the actual climatic situation, the mentioned long-term studies, as the one described here, are of great importance in the evaluation of pollution levels in areas far from emission sources in order to verify the real decrease in the concentrations of the main pollutants, particularly PM_10_, as a result of the application of European emission reduction policies and plans of regional and local proposals by public environmental authorities.

Therefore, the present study focuses on the analysis of the daily PM_10_ values as a means of finding the relationship between weather types and PM_10_ concentration in several background stations located in mainland Spain from 2001 to 2019. The temporal trend of PM_10_ has been analyzed during this period, and a cluster analysis has been carried out to group air quality stations with similar PM_10_ concentrations. Finally, the relationship between PM_10_ exceedances and weather types has also been evaluated.

## 2. Study Site

**Figure 1:**
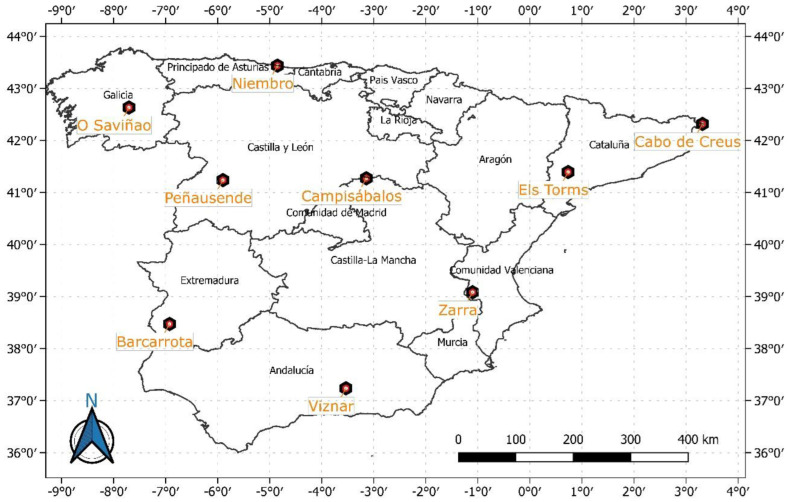
Location of the background stations used in the study. Administrative boundaries’ layer retrieved from IGN (https://www.ign.es/web/rcc-info-delimitaciones (accessed on 1 September 2022)) and modified through QGIS.

The study area covers the whole of mainland Spain through nine air quality stations [29] belonging to the Spanish network EMEP/VAG/CAMP between 2001 and 2019. All the stations analyzed in the study are background stations located far away from any possible anthropogenic source of pollution (Table 1, Figure 1). The geographic distribution of the stations is rather different. Four of them are not far from the Mediterranean coast (Cabo de Creus, Els Torms, Zarra, and Víznar), two of them in the north (Cabo de Creus and Els Torms) and the other ones in the south. Niembro is located in the Cantabrian coast, while the remaining four stations (O Saviñao, Peñausende, Campisábalos, and Barcarrota) could be considered inland stations. In mainland Spain, most of the time, the effects of the North Atlantic anticyclone cause local thermal circulations to appear, such as coastal or mountain breezes [30]. On the other hand, the proximity with the north of Africa and Central and Eastern Europe makes particle-laden air masses come into the peninsula through these areas, which could be considered a potential source of anthropogenic pollution, particularly in the cases of Central and Eastern Europe [31], or natural pollution as in the case of Saharan dust intrusions from Northern Africa [32]. In addition, in the Mediterranean coast, where four stations are located (Figure 1), breeze episodes could favor the dispersion of pollutants during most of the year [33].

## 3. Materials and Methods

### 3.1. Databases

Daily PM_10_ data were obtained from the EBAS website (http://ebas.nilu.no/ (accessed on 2 September 2022)), developed and operated by the Norwegian Institute for Air Research (NILU). Information related to European stations used in the study (location, instrumentation, etc.) is retrieved from the EMEP website. All the stations are equipped with the same instrumentation, and the data are collected and analyzed following the same protocol established by the EMEP [34]. Daily data were collected at 0700 UTC and sent once a week to the Carlos III Health Institute, to the Atmospheric Pollution Department. A high-volume collector was used for PM_10_ samplings, and its concentration determined by the gravimetric method.

The daily data of sea level pressure used to determine the weather types have been obtained from the website of the National Center for Atmospheric Research, available at https://ncar.ucar.edu/ (accessed on 2 September 2022).

### 3.2. Data Analysis

Two different statistical analyses were applied. First, a univariate analysis for describing the data and their distribution by calculating kurtosis, standard deviation, skewness, minimum, maximum, median, and variance (based on daily data). Second, a multivariate analysis to find similarities between stations by grouping them according to similar PM_10_ values. This analysis was carried out using MATLAB to obtain a dendrogram, following the Ward aggregation method jointly with the Euclidean distance [35]. The smaller distances indicate a greater relationship between stations grouped in the same cluster [36,37].

### 3.3. Lamb Classification in Weather Types

The Lamb classification evaluates the daily circulation patterns in a way to find a method able to classify them. The methodology followed by Jenkinson and Collinson [38] and Jones et al. [13] to objectively define different weather types in the British Isles, and based on the Lamb classification, has been applied in mainland Spain. In this area, the application of this classification was developed by Spellman [10] and Trigo and DaCamara [11], who established the location of the network of points in the Iberian Peninsula. The application relies on the determination of several indices associated with the direction and vorticity of the geostrophic flux [39], resulting in a classification into 26 weather types, as shown in Table 2. For the whole study period, every single day was characterized by a specific weather type. This classification of the synoptic conditions was then used to find a possible relationship between PM_10_ concentrations and weather types.

### 3.4. Regulations, Limit Values, and Intrusions

For each station, daily values of PM_10_ concentration exceeding the daily limit value of 50 µg m^−3^ established by European directives were identified. Thanks to the information included in the annual reports provided by the Ministry for Ecological Transition and Demographic Challenge regarding to the identification of natural episodes of transboundary contributions of particles (African episodes), and other types of natural episodes, the previously identified days exceeding the daily PM_10_ limit value have been linked or not to Saharan intrusion episodes. The methodology followed in those reports to identify days related to intrusion episodes coming from the Sahara desert, applying the HYSPLIT model together with other tools, is widely described at https://www.miteco.gob.es/es/calidad-y-evaluacion-ambiental/temas/atmosfera-y-calidad-del-aire/metodologiaparaepisodiosnaturales-revabril2013_tcm30-186522.pdf (accessed on 18 October 2022).

## 4. Results and Discussion

### 4.1. Analysis of the Representative Values for Each Station

Results for the univariate statistical analysis of PM_10_ data are shown in Table 3. Víznar was the station with the lowest missing data for the whole period in contrast to Campisábalos, which presented almost 20% of missing data. Among all the stations included in the study, Cabo de Creus, Niembro, and Víznar stood out for presenting high PM_10_ mean concentrations, with around 17 µg m^−3^ or higher. Minimum averaged values were obtained for most of the stations located inland, such as Campisábalos, O Saviñao, and Peñausende. The highest daily PM_10_ concentration was reached in Zarra, with 320 µg m^−3^. However, this station was not the one presenting the highest mean concentration, which was recorded in Víznar. This fact proves that maximum values can greatly differ from mean values due to isolated episodes of high concentrations. Mean values are also affected by those isolated episodes of high concentrations, something that is corroborated by the high values of the standard deviation shown in Table 3. Therefore, median values better represent characteristic concentration values for each station. Stations presenting high maximum values were all located in the southern area of the peninsula (Zarra, Víznar, and Barcarrota). All stations present similar positive skewness values, which means all distributions are right-skewed. Similarly, all stations present positive kurtosis values (Table 3). This means that the concentrations registered are closely grouped together around a narrow interval close to the median.

**Figure 2:**
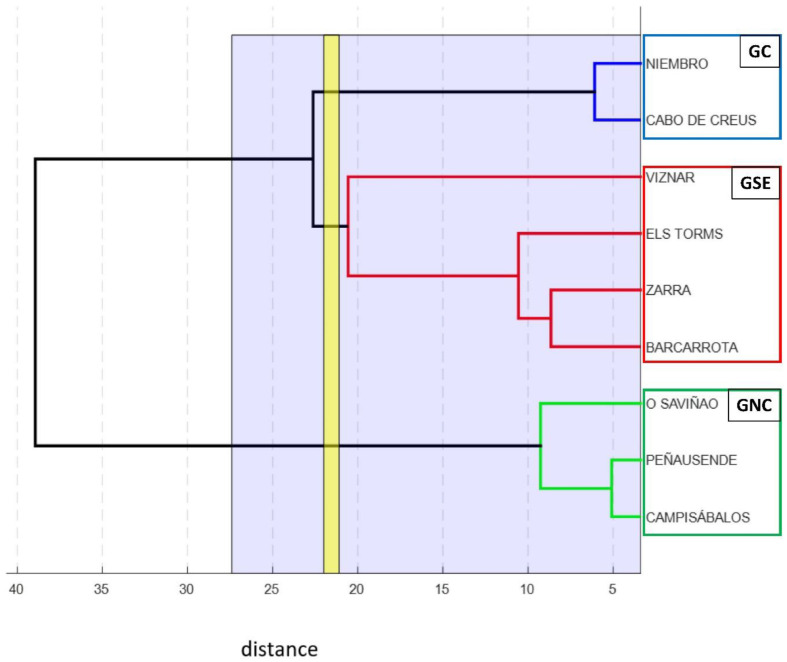
Dendrogram showing the clusters regarding similarities in PM_10_ monthly concentration values.

Results of the multivariate statistical analysis led to the clustering shown in the dendrogram in Figure 2. Average values for each month and station were taken into consideration in the dendrogram. Finally, three main groups of stations located in the coastal (Cabo de Creus and Niembro), north–central (Campisábalos, O Saviñao, and Peñausende), and southeastern (Barcarrota, Els Torms, Víznar, and Zarra) areas were obtained. For later references, these groups were named as GC (coastal), GNC (north–central), and GSE (southeastern). The results of the dendrogram vary considerably, depending on the input values that are used for the calculation. Results for dendrogram tests with different input values are not shown in this paper. However, there are three stations that always remain in the same cluster regardless of whether the input values were monthly or yearly or included other types of pollutants. These stations are Campisábalos, O Saviñao, and Peñausende. As seen in Table 1 and Figure 1, Niembro and Cabo de Creus are the stations nearest to the coast and characterized by the minimum altitude, their location being almost at sea level.

### 4.2. Monthly and Annual Evolution

**Figure 3:**
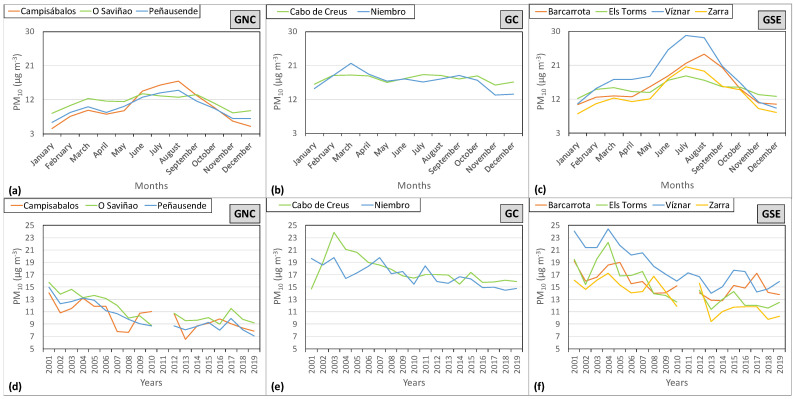
Monthly (**a**–**c**) and yearly (**d**–**f**) evolution of PM_10_ for the clustering group and station for the study period 2001–2019.

Figure 3 shows the monthly and yearly evolution of PM_10_ for the different groups established. This evolution is markedly different between the different groups of stations. Figure 3b, where GC is represented, shows a practically constant value during all the months with a slight decrease in winter. In contrast, GNC (Figure 3a) and GSE (Figure 3c) present a monthly evolution with a slight increase in the concentrations from January to March, followed by a slight decrease in April, which gives way to a continuous increase from May to July/August when the maximum value is reached. The slight decrease in April can be explained by the increasing frequencies of the advections of Atlantic air masses associated with high rates of precipitation [30] during this month.

Considering a common range for all the groups (Figure 3) covering from 3 to 30 µg m^−3^, for the monthly evolution, GNC (Figure 3a) maintains its values practically within the first third range, GC (Figure 3b) presents almost a constant monthly evolution with values corresponding to the second third, and GSE (Figure 3c) covers the entire range of values. GNC and GSE present minimum values in winter and maximum ones in summer. There is a clear difference between them, the maximum value (with the exceptions of O Saviñao and Barcarrota) is reached in August for GNC and in July for GSE, reaching maximum values greater than those stations belonging to GSE, particularly Víznar with a value of 28.97 µg m^−3^ in July.

PM_10_ concentrations are closely related to meteorological conditions. During summer, when the maximum concentration is reached, anticyclonic situations are frequent with a low capacity of air mass renewal, and consequently, a low dispersion of pollutants is registered. These conditions also favor the resuspension of soil particles. During winter, these conditions are much less frequent, and therefore, the mean concentrations are much lower [30]. Besides, the intrusions of Saharan dust notably affect the stations located in the south and east of the Iberian Peninsula. There is a special incidence of intrusions during summer [40,41], and this must be taken into account when analyzing the results obtained and presented in Figure 3.

**Figure 4:**
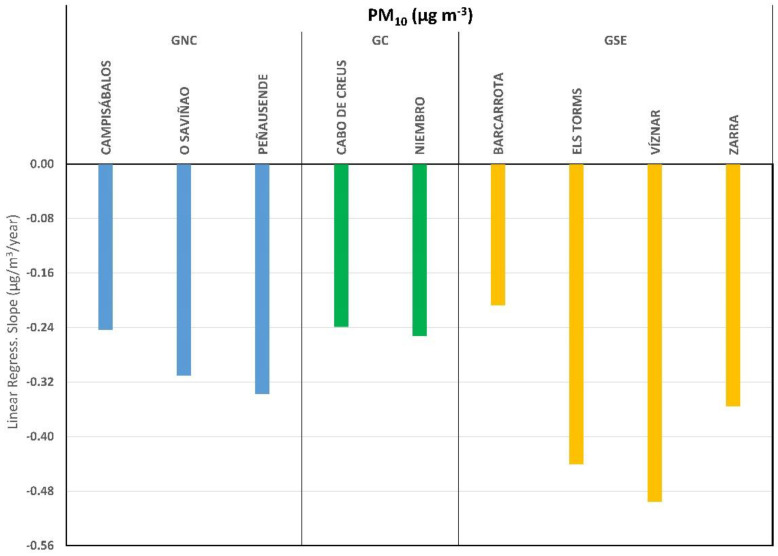
Annual trends by clustering group and station for the study period 2001–2019.

Annual averaged PM_10_ values were calculated for each station and the whole study period, and the results are shown in Figure 3d–f. The differences between groups rely on the concentration levels, GNC being the one with a lower level, as mentioned above (Figure 3a–c). Although the trend has been downward, some stations have increased their concentrations in the past few years, particularly the stations in GSE. The quantification of these downward trends has been computed using a linear regression (Figure 4). Decreasing rates seem not to be related to the established groups, and differences could be found between stations even in the same group. All stations have shown a statistically significant decreasing trend in their PM_10_ concentrations with values for the determination coefficient (R^2^) from 0.35 to 0.78 for Cabo de Creus and Peñausende, respectively.

Figure 4 presents the stations alphabetically ordered within their group. Even within the same group, not all stations show the same trend in magnitude, and it should be noted that a common behavior cannot be found when analyzing decreasing trends. The values show that the station with the strongest trend is Víznar with a decrease of approximately 0.5 µg m^−3^ per year, followed by Els Torms and Zarra, all of them in the same group (GSE). Similar results were obtained by Querol et al. [26] in the same EMEP stations for a study period between 2001 and 2012.

In the Iberian Peninsula, PM_10_ is generally more affected by African intrusions, while PM_2.5_ is more affected by anthropogenic emissions into the atmosphere [26]. Therefore, the application of European strategies to reduce pollution, as well as the impact of the financial crisis on southern Europe that has originated a sharp decrease in pollutant emissions [42,43], could have more impact on PM_2.5_ concentrations than on PM_10_ concentrations, even when the decreasing rates for PM_10_ are quite high (Figure 4).

### 4.3. PM_10_ and Regulations

Taking into consideration the threshold set by the European directives and regulations, for the study period of 19 years, none of the nine stations surpassed the value of 35 days a year in the PM_10_ concentration case. The maximum number of days surpassing that value was around 10 for almost half the stations, with the exception of Peñausende and O Saviñao (both in GSE) with lower values. Only two stations, Els Torms and Víznar, registered more days with values over 50 µg m^−3^, 15 and 25 days, respectively, in 2003. When considering the whole study period, Víznar stood out as the station with the highest number of days surpassing the threshold value (196 days), far from the ones registered in the other stations. However, its mean concentration during these days remains similar to the ones registered in the other stations with a value of 73.9 µg m^−3^. On the other hand, the station with the smallest number of exceedances was O Saviñao with 25 days in the total study period and a mean concentration of 71.9 µg m^−3^, followed by Cabo de Creus, with 38 days surpassing the daily threshold value, and with a mean concentration of 66.9 µg m^−3^.

**Figure 5:**
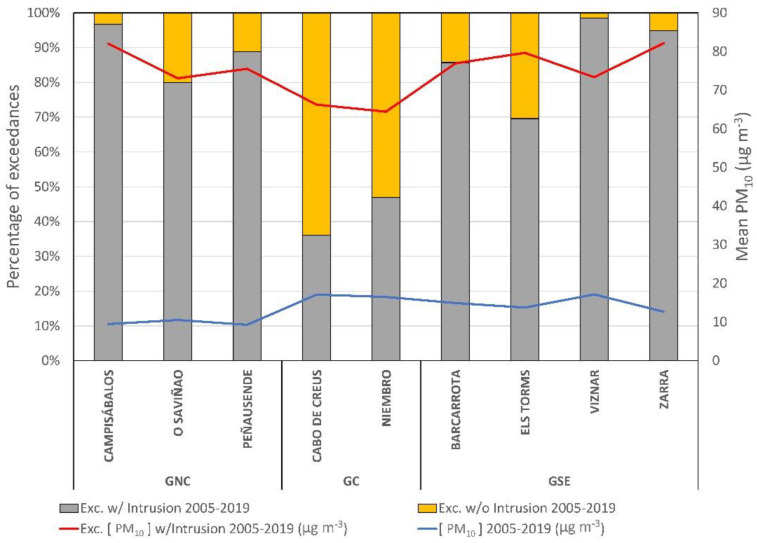
Relationship between the percentage of days exceeding the daily threshold value related (gray column) or not (orange column) with Saharan dust intrusions for the period 2005–2019. Mean PM_10_ concentrations for the days exceeding the daily threshold value for the whole measuring period (blue line) and for the values related to intrusions (red line) are also shown.

In the background stations used for this study, the anthropogenic influence is minimal, so the isolated cases when the daily PM_10_ concentration threshold was surpassed could be related to Saharan dust intrusions [32] or to the presence of PM coming from Central Europe [44]. Consequently, the episodes of Saharan dust intrusions were studied, as they constitute one of the main causes of the exceedances in the daily threshold value. Figure 5 presents the percentage of exceedances related to intrusions in each station for the period 2005–2019. Only this period has been analyzed due to the lack of data from 2001 to 2004. For the 2005–2019 period, both exceedances and intrusions data were available in the URL of the Spanish Ministry for Ecological Transition and Demographic Challenge [45].

The mean PM_10_ concentrations computed for the total sampling period or only for the days exceeding the daily threshold value greatly differ, and results are also shown in Figure 5. For the total period, mean PM_10_ ranged between 9.3 and 17.2 µg m^−3^, with small differences among stations. However, in the case of exceedances, PM_10_ concentration soared, reaching values of 66.2 to 82.1 µg m^−3^. Complementarily, the analysis of the exceedances of the threshold value combined with the episodes of intrusions has been carried out. Except in the case of the stations belonging to GC, in the rest of the cases, it has been observed that the exceedances are highly linked to episodes of Saharan dust intrusions (Figure 5). Particularly, stations located in the south of mainland Spain, such as Víznar and Zarra (Figure 5), were greatly affected by those Saharan intrusions [46]. Two clear exceptions can be seen, Cabo de Creus and Niembro, both in GC, for which over 50% of the exceedances were not related to episodes of Saharan dust intrusions (Figure 5). It is in these two stations where the average values (with daily PM_10_ over 50 µg m^−3^) were also lower than in the rest of the stations (red line in Figure 5).

### 4.4. Relationship between PM_10_ and Weather Types

**Figure 6:**
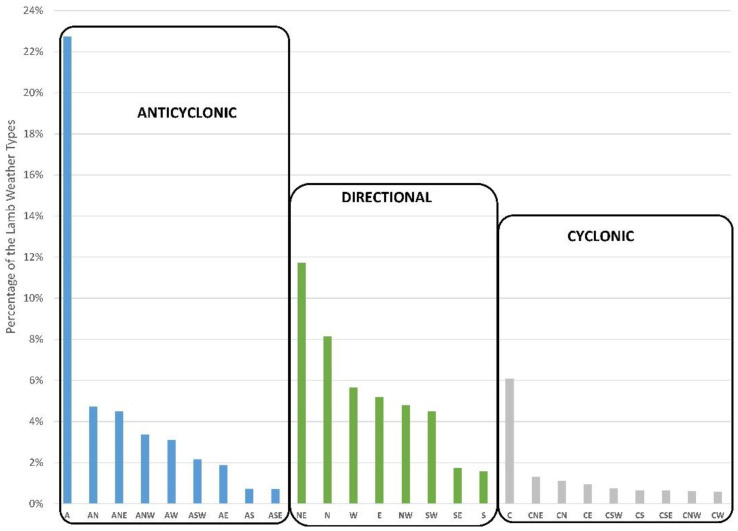
Frequency of each weather type for the period 2001–2019.

Figure 6 shows the total frequency of the Lamb weather types for the period 2001–2019 in the study area. The different types are classified into three main groups: anticyclonic, directional, and cyclonic. The prevalent type is A, accounting 22% of the total for the whole study period, followed by NE and N types, with 12% and 8%, respectively. The most dominant weather type in the area is, thus, the A type, followed by almost all directional types. Cyclonic types are less frequent, with frequencies below 2%, with the exception of the C type.

In this study, the three most frequent weather types (A, NE, and N) are characterized by atmospheric stability. A similar result was obtained by Grimalt et al. [47], who studied the distribution of the weather types in the Mediterranean basin, where several of the stations studied in this paper are located, finding out as a result that the most frequent type was A, followed by C. The weather types depend on various meteorological parameters and help us study their influence on the concentration of pollutants. Among all those types, for example, the anticyclonic is the one that originates the most relevant pollution scenarios [48], being the weather type prevailing in the Iberian Peninsula during the study period (Figure 6).

**Figure 7:**
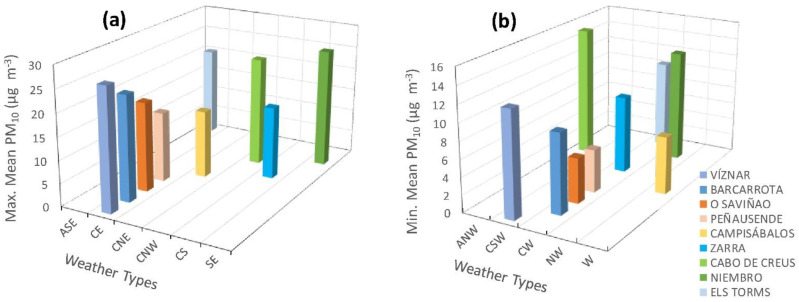
Distribution of the weather types related to maximum (**a**) and minimum (**b**) mean PM_10_ for the period 2005–2019. Each color indicates a single station. Only weather types with the maximum or minimum values have been represented.

The results of the analysis of the relationship between PM_10_ concentrations and weather types are shown in Figure 7. This figure shows the maximum and minimum averaged concentration values for each station and weather type. Mean PM_10_ values were calculated for every weather type and station for the period 2005–2019, and maximum and minimum mean PM_10_ values were identified for every station. Only weather types corresponding to some maximum or minimum value are presented in the graphs (Figure 7). Almost half of the highest concentrations were measured under CE conditions, with the remaining ones a bit more dispersed between different weather types, depending on the station. The weather type that presents the minimum PM_10_ mean concentration is the CW type for more than half of the stations except for those in GC: Niembro (NW type) and Cabo de Creus (ANW). The other two exceptions are Campisábalos (W) and Víznar (CSW). This result shows that minimum and maximum mean concentrations tend to concentrate around the same weather type.

Spellman [10] states that the C-type during summer is related to low pressure systems coming from central Sahara. However, the results presented in Figure 7 show that the C type does not stand out in any station as one of the types where maximum or minimum concentrations were measured.

**Figure 8:**
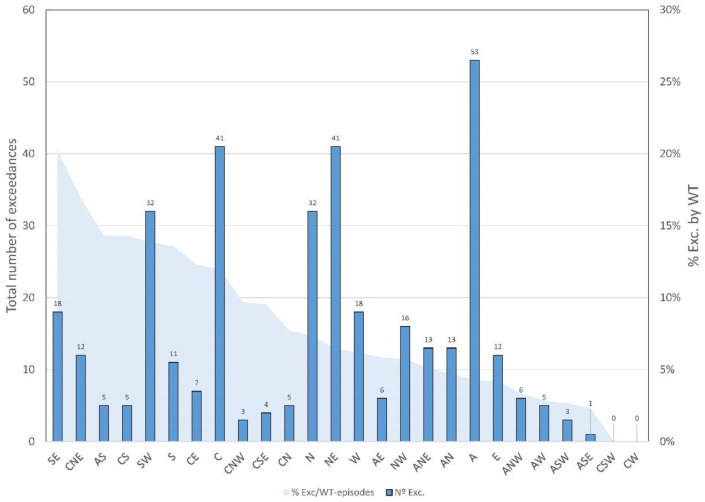
Total number of exceedances (PM_10_ > 50 µg m^−3^) for each weather type in the period 2005–2019, and ratio (in percentage) between the number of exceedances and the total number of episodes for each weather type.

A detailed analysis of the exceedances in the daily PM_10_ concentration (>50 μg m^−3^) has also been carried out, considering the relationship between those exceedances and the weather types. The cases where the exceedances are linked to Saharan dust intrusions have also been investigated. Figure 8 shows the relationship between the number of episodes for each weather type and the total number of PM_10_ exceedances during the whole study period. As in the results shown in Figure 6, the weather type with the highest number of episodes is A, followed by the NE, C, SW, and N types with values of 53, 41, 41, 32, and 32, respectively. Since the number of episodes related to each weather type is not the same, the fact that the A type is the one that presents the greatest number of exceedances is not indicative of a strong linkage to PM_10_ exceedances. Because A is the most frequent type, it was expected to present the highest number of exceedances. For this reason, an intercomparison of the weight of each weather type against the number of exceedances and the PM_10_ concentration must be performed.

Taking into consideration the real weight of each weather type in the exceedance episodes, a more real representativeness of each weather type related to PM_10_ exceedances can be obtained. Figure 8 shows the ratio between the exceedances and the frequency of weather types (blue area). The type with the highest normalized number of exceedances is SE, stating that 20.2% of the episodes of this weather type were associated with exceedances of the daily PM_10_. The A type is very far from this value, with less than 5% of the episodes of this weather type related to PM_10_ exceedances. It should be noted that there are three weather types that have presented one or no exceedance at all during the whole study period: ASE, CSW, and CW. There are other weather types that present similar characteristics, but still present exceedances and a high ratio when the normalized values are analyzed, as shown in Figure 8 (CNW, AS, CS, and CSE). Similar results for the weather types related to C have been obtained by other authors when the relationship between this type and rainfall has been studied [11]. Fernández-González et al. [7] also studied the relationship between weather and precipitation, stating that the C, W, and SW types provide more rainfall than the remaining types.

### 4.5. Relationship between PM_10_ and Other Variables

First, an analysis seeking a possible relationship between PM_10_ and altitude, longitude, and latitude was carried out. For the period 2005–2019, the number of PM_10_ exceedances of the daily threshold value was counted, and the mean PM_10_ concentrations above that limit were computed for each air quality station, only for episodes of Saharan dust intrusions. In the case of longitude, no correlation was found between these variables (R^2^ < 0.03). However, latitude and altitude presented a correlation with the number of days of exceedances (R^2^ = 0.68) and with the average PM_10_ (without intrusion) concentrations (R^2^ = 0.44), respectively, both statistically significant for a significance level of 0.05. The correlation found is positive in the case of altitude (slope = +0.01 µg m^−3^/m.a.s.l.) and negative in the case of latitude (slope = −14.73 number of exceedances with intrusion/deg.), which seems coherent: the greater the distance from the Sahara, the lower the frequency of intrusions.

**Figure 9:**
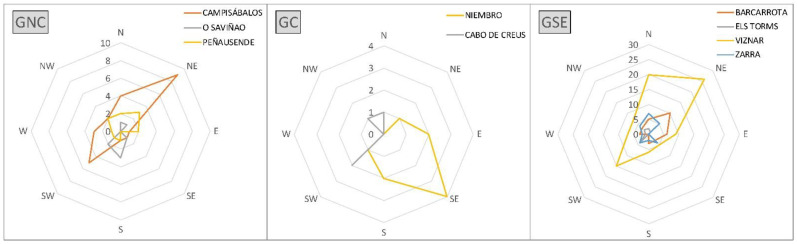
Wind roses showing the number of PM_10_ exceedances related to Saharan intrusions for the period 2005–2019.

Second, the relationship between wind direction and PM_10_ was analyzed. The various weather types classified in Table 2 were grouped according to the prevailing wind direction into eight categories, excluding the A and C types. The number of exceedances under the presence of an intrusion episode was related to those categories, and the results are shown in Figure 9. For the stations belonging to GC, there is no common prevailing direction related to exceedances of the PM_10_ daily threshold value. However, for GSE and GNC, the NE–SW direction seems to be the prevailing direction, although for GSE, also the weather types characterized by a north component are related to exceedance episodes, especially in Víznar.

## 5. Conclusions

A full analysis of PM_10_ has been made for nine background EMEP stations covering the whole of mainland Spain. Three groups have been established, clustering the stations with similar characteristics and pollutant concentrations using a dendrogram: GC (coastal location), GNC (north–central location), and GSE (south–eastern location).

The lowest mean concentrations were registered for stations in GNC, and the highest concentrations were registered in stations belonging to the GSE group. Maximum values for PM_10_ were reached during summer, mainly influenced by meteorological conditions and Saharan dust intrusion episodes. In addition, a constant negative trend was found for each station analyzed from the beginning of the data series in 2001 until 2019.

The two stations that stand out for presenting the highest number of exceedances related to intrusions were Campisábalos and Víznar. In contrast, the two stations with the highest number of exceedances without direct relation to an intrusion were Cabo de Creus and Niembro.

The prevailing weather type in mainland Spain is anticyclonic; as a result, this is the weather type presenting the highest number of exceedances. Since it is the dominant weather type, it is logical to expect that it will be the type that also presents the highest number of exceedances. Despite this, the anticyclonic weather type is not the one characterized by most exceedances. When taking into consideration the weight of every weather type regarding the total of measurements, it is the southeasterly directional type, the one for which every episode is related to exceedances, followed by the cyclonic northeasterly type.

When computing mean PM_10_ concentrations for every weather type and station, results state that minimum and maximum mean concentrations tend to concentrate around the same weather type, cyclonic westerly and cyclonic easterly, respectively.

## Figures and Tables

**Table 1:** Features of the background stations belonging to the network EMEP/VAG/CAMP and used in the study.

No.	National Cod.	International Cod.	Name	Region	Latitude (deg.)	Longitude (deg.)	Altitude (m a.s.l)
1	33036999	ES0008R	Niembro	Asturias	43.439	−4.850	134
2	27058999	ES0016R	O Saviñao	Lugo	42.635	−7.705	506
3	17032999	ES0010R	Cabo de Creus	Girona	42.319	3.316	23
4	25224999	ES0014R	Els Torms	Lleida	41.394	0.735	470
5	19061999	ES0009R	Campisábalos	Guadalajara	41.274	−3.143	1360
6	49149999	ES0013R	Peñausende	Zamora	41.239	−5.898	985
7	46263999	ES0012R	Zarra	Valencia	39.083	−1.101	885
8	06016999	ES0011R	Barcarrota	Badajoz	38.473	−6.924	393
9	18189999	ES0007R	Víznar	Granada	37.237	−3.534	1230

**Table 2:** Lamb classification of weather types.

Weather Types					
Anticyclonic		Directional		Cyclonic	
A	Anticyclonic			C	Cyclonic
ANE	Anticyclonic northeasterly	NE	Northeasterly	CNE	Cyclonic northeasterly
AE	Anticyclonic easterly	E	Easterly	CE	Cyclonic easterly
ASE	Anticyclonic southeasterly	S	Southerly	CSE	Cyclonic southeasterly
AS	Anticyclonic southerly	SE	Southeasterly	CS	Cyclonic southerly
ASW	Anticyclonic southwesterly	SW	Southwesterly	CSW	Cyclonic southwesterly
AW	Anticyclonic westerly	W	Westerly	CW	Cyclonic westerly
ANW	Anticyclonic northwesterly	NW	Northwesterly	CNW	Cyclonic northwesterly
AN	Anticyclonic northerly	N	Northerly	CN	Cyclonic northerly

**Table 3:** Averaged concentration (±standard deviation), maximum, minimum, median, kurtosis, skewness, and variance values of the data series covering the period from 2001 to 2019 for each weather station. Percentages of missing data per station are also shown.

	*STATION*	*BARCARROTA*	*CABO DE CREUS*	*CAMPISÁBALOS*	*ELS TORMS*	*NIEMBRO*	*O SAVIÑAO*	*PEÑAUSENDE*	*VÍZNAR*	*ZARRA*
*POLLUTANT*										
**PM_10_ (μg m^−3^)**		**15.47 ± 11.48**	**17.54 ± 8.20**	**10.11 ± 10.09**	**14.89 ± 9.75**	**16.90 ± 9.27**	**11.35 ± 8.16**	**10.13 ± 8.75**	**18.31 ± 15.32**	**13.39 ± 10.75**
*Maximum*		*246*	*133*	*200*	*169*	*104*	*157*	*197*	*309*	*320*
*Minimum*		*0*	*0*	*0.5*	*2.0*	*1.0*	*1.0*	*1.0*	*1.0*	*1.0*
*Median*		*12*	*16*	*8*	*13*	*15*	*9*	*8*	*15*	*11*
*Kurtosis*		*52.38*	*18.46*	*51.86*	*23.35*	*6.76*	*33.48*	*61.85*	*44.29*	*126.45*
*Skewness*		*4.64*	*2.56*	*4.90*	*3.16*	*1.84*	*3.78*	*5.11*	*4.33*	*6.62*
*Variance*		*131.80*	*67.28*	*101.82*	*95.15*	*85.94*	*66.61*	*76.48*	*234.96*	*115.64*
*% Missing Values*		*13.3%*	*9.6%*	*18.5%*	*12.6%*	*10.5%*	*14.8%*	*12.7%*	*7.2%*	*9.5%*

## Data Availability

The results presented in this study have been processed from the data available on the websites mentioned in the main text.
